# News and Miscellany

**Published:** 1902-02

**Authors:** 


### News and Miscellany.

**Dr. L. L. Loggins** requests us to say that he is permanently lo. cated to practice medicine at Willis, Montgomery county, Texas.

**Lee A. Loggins, M D.,** Washington University, St. Louis, 1898, a practitioner of Graham, Texas, died at the house of his father, Dr. J. C. Loggins, in Ennis, Texas, December 27.

**Doctor,** I will sell my eye and ear practice here which is paying over $500.00 a month. Business new, clean and up to date. If you can handle the work and are looking for something in this line, it will pay you to investigate. Dr. SEYMOUR, 1013 Texas Avenue, Houston, Texas.

**New Orleans Polyclinic** now in session, 15th year. Closes May 31, 1902.—Physicians will find the Polyclinic an excellent means for posting themselves upon modern progress in all branches of medicine and surgery. The specialties are fully taught, particularly laboratory work. For further information address Dr. Isadore Dyer, Secretary, New Orleans Polyclinic, postoffice box 797, New Orleans, La.

---

**The following Texas physicians** were in attendance at the New Orleans Polyclinic, January 1, 1902: Dr. I. T. Clemons, Monaville; Dr. B. E. Hudgins, Crandell; Dr. W. T. Brown, Walls; Dr. Oscar D. Radney, Mount Calm; Dr. J. M. Blackwell, San Antonio; Dr. M. W. Pitts, Rosenberg; Dr. J. M. Watkins, Luling; Dr. H. R. Ammons, Turnersville; Dr. J. B. Gordon, Paluxy; Dr. W. G. Elliott, West; Dr. W. E. Wills, Tours.

---

**Ecthol in Smallpox.** Battle & Co., St. Louis, manufacturers of the great anti-purulent, ecthol, have issued a small brochure on smallpox, written by Dr. J. A. Knight, of Eatonville, Ga. It gives much valuable information as to the clinical history, progress, complications of the disease, and the points in differential diagnosis, also the treatment. The writer strongly recommends ecthol. A postal card to Battle & Co., the well-known Bromidia men, St. Louis, mentioning the "Red Back," will fetch a copy of the pamphlet, instanter.

---

**The** *American Gynecological and Obstetrical Journal*, so long and ably conducted by Dr. J. Duncan Emmet, 91 Madison Ave., New York, we greatly regret to learn has discontinued publication. We hope that it will be a temporary suspension only, and that we may soon be gladdened by its reappearance on our desk. It was one of the cleanest, ablest, most dignified and high toned journals on our exchange list, and we shall miss it. We learn from *American Medicine* that the cause of suspension was the neglect of over "five thousand physicians to pay their subscription!" Shame! Shame! They do better than that in Texas, and the "Red Back" is proud of its subscribers. *American Medicine* says, in speaking of Dr. Emmet's loss: "But the lessons to every honorable member of our guild should be taken to heart and should result in immediate action:

"1. Subscribe for journals owned by physicians and conducted solely for professional interests.

"2. Work with your colleagues and friends to this end.

"3. Pay your subscriptions promptly.

"In this way only can professional unity and reform be secured."

**Dr. Lott's paper** on "The Drug Habit: Its Treatment," was published in this journal for November, 1901. It was read at the Calvert meeting of the Brazos Valley Association. Since its publication Dr. Lott has received so many letters from physicians asking if he would receive such cases for treatment that he takes this method of announcing that he is prepared to and will receive and treat cases of opium, cocaine, chloral or whiskey addiction by the hyoscine treatment as described in the paper referred to. The method is well known to the readers of this journal. It is speedy and painless. Physicians who do not care to treat these cases, or who are not prepared to do so, are invited to correspond with him, and can send them to him with every assurance that they will be in good hands and will be treated strictly in accordance with ethical principles. They will be cared for in a private family residence, where Dr. Lott will personally treat them, and will be in the charge of skilled, trained and experienced nurses. Every comfort and convenience will be furnished, in the privacy of a home (not a sanitarium), including good family fare, and cooking—privacy, baths, etc. Ladies will be received as well as men, and will have the services and constant attention of trained lady-nurses. Dr. Lott refers by permission to the editor of the Texas Medical Journal. Address Dr. M. K. Lott, Cameron, Texas.

